# Supplementary material for: Venomix: a simple bioinformatic pipeline for identifying and characterizing toxin gene candidates from transcriptomic data
Source: PeerJ. 2018 Jul 31;6:e5361. doi: 10.7717/peerj.5361 (PMC6074769; doi:10.7717/peerj.5361)
Supplement: Supplemental Information 6 [file peerj-06-5361-s006.gz › FinalOutput_1E-6/Zinc_metalloproteinase@disintegrin_5/finaltree.pdf]

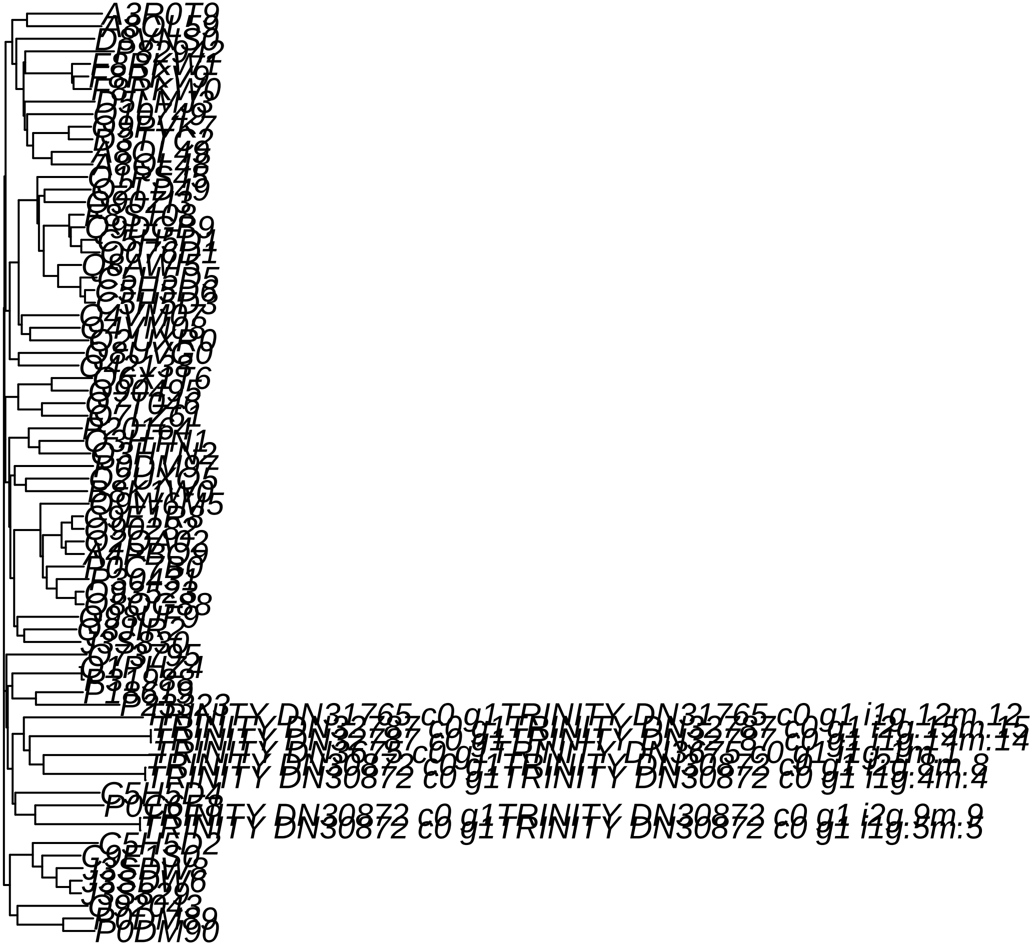
A3R0T9
TRINITY DN31765 c0 g1TRINITY DN31765 c0 g1 i1g.12m.12
TRINITY DN30872 c0 g1TRINITY DN30872 c0 g1 i1g.4m.4
TRINITY DN30872 c0 g1TRINITY DN30872 c0 g1 i2g.9m.9
TRINITY DN30872 c0 g1TRINITY DN30872 c0 g1 i1g.3m.5
P0DM90
